# Short-term effects of various non-steroidal anti-inflammatory drugs (NSAIDs) on *Danio rerio* embryos

**DOI:** 10.1016/j.mex.2023.102215

**Published:** 2023-05-11

**Authors:** Imen Ben Chabchoubi, Rim Attya Bouchhima, Nacim Louhichi, Aissette Baanannou, Saber Masmoudi, Olfa Hentati

**Affiliations:** a Institut Supérieur de Biotechnologie de Monastir (ISBM), Rue Taher Haddad, 5000, University of Monastir, Monastir, Tunisia; b Laboratoire Génie de l'Environnement et Ecotechnologie (GEET), Ecole Nationale d'Ingénieurs de Sfax (ENIS), Route de Soukra, Km 4, 3038, University of Sfax, Sfax, Tunisia; c Unité Cibles pour le Diagnostic et la Thérapie, Route Sidi Mansour, Km 6, 3018, Sfax, Center of Biotechnology of Sfax (CBS), Tunisia; d Institut Supérieur de Biotechnologie de Sfax (ISBS), Route de Soukra, Km 4, 3038, University of Sfax, Sfax, Tunisia

**Keywords:** Fish embryo toxicity (FET) test, Environmental risk assessment, Risk quotient, Toxic unit, Determine the toxicity and environmental risks of diclofenac, ibuprofen, ketoprofen, and paracetamol in zebrafish embryos

## Abstract

Due to the widespread use of non-steroidal anti-inflammatory drugs (NSAIDs) without a medical prescription and their frequent prevalence in aquatic habitats, there are major health and environmental issues. NSAIDs have been found in surface water and wastewater in concentrations ranging from ng/L to μg/L all over the world. The purpose of this study was to determine the relationship between NSAIDs (diclofenac, ketoprofen, paracetamol and ibuprofen) exposure and associated adverse effects in the assessment of indirect human health risks posed by *Danio rerio* (zebrafish) and Environmental Risk Assessment (ERA) of these NSAIDs in aquatic environments. Therefore, the objectives of this study were to (i) reveal abnormality endpoints of early developmental stages, after exposure of zebrafish and (ii) perform an ecological risk assessment of aquatic organisms upon exposure to NSAIDs detected in surface waters based on the risk quotients (RQs) method. According to the toxicity data collected, all of the malformations appeared after diclofenac exposure at all concentrations. The most notable malformations were the lack of pigmentation and an increase in yolk sac volume, with EC_50_ values of 0.6 and 1.03 mg/L, respectively. The results obtained for the ERA revealed RQs higher than 1 for all the four NSAIDs chosen, posing ecotoxicological pressure in aquatic environments. Overall, our findings provide a critical contribution to the formulation of high-priority actions, sustainable strategies and strict regulations that minimize the negative effects of NSAIDs on the aquatic ecosystem.

• To determine the LC_50_, lethal conditions such as coagulation, absence of heartbeat and blood flow, absence of tail separation and development of somites were taken into account.
• The EC_50_ was calculated using sublethal parameters such as blood coagulation, pericardial edema, yolk sac edema or hypertrophy.
• The 4 compounds present a high risk individually and in mixture with a RQ >> 1.

## Graphical abstract

**Figure:**
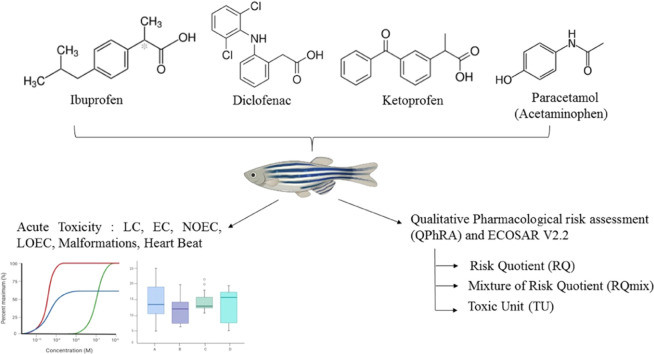

Conclusion : All four NSAIDs are found to be high risk compound with a RQ >> 1. Therefore, there is a need for strict regulation of the release of NSAIDs into environmental media to minimize their toxicity to aquatic organisms.

Specifications table

**Table:** 

Subject area:	Pharmacology, Toxicology and Pharmaceutical Science
More specific subject area:	*NAIDs disruption in the fish cycle Danio rerio (5 dpf)*
Name of your method:	Determine the toxicity and environmental risks of diclofenac, ibuprofen, ketoprofen, and paracetamol in zebrafish embryos
Name and reference of original method:	*OECD Guidelines for the Testing of Chemicals. Section 2: Effects on Biotic Systems Test No. 236: Fish Embryo Acute Toxicity (FET) Test. Paris, France: Organization for Economic Cooperation and Development., (2013)*
Resource availability:	*Test No.236: Fish embryo Acute toxicity (FET) Test* https://ecosar.software.informer.com/

## Introduction

Pharmaceutical active substances have been found in a variety of aquatic habitats, including surface water, lakes, rivers, streams, estuaries, and seawater [1], [2], [3], [4], [5]. The NSAIDs have been shown to be pharmacologically active, resistant to degradation, persistent in aquatic systems, and damaging to non-target creatures such planktons [6], mollusks [7], and fishes [8], [9], [10]. The main source of NAIDs is the excretion of unfed drugs in urine and feces, but other sources include unused drug disposal in toilets, as well as industrial and hospital discharges. In this case, the NSAIDs are transported to WWTPs, where some can be removed by traditional secondary treatment but many cannot be treated and are discharged into surface waters, where they can escape and spread to groundwater and even to the ocean [11], [12], [13]. According to Kummerer [14], low removal rates from WWTPs result in the release of a mixture of pharmaceuticals and metabolites to receiving water bodies. The inability of standard treatments to completely remove NSAIDs residues from plant influent is most likely the major cause of their presence in treated wastewaters [15], [16], [17], [18].

**Fig. 1:**
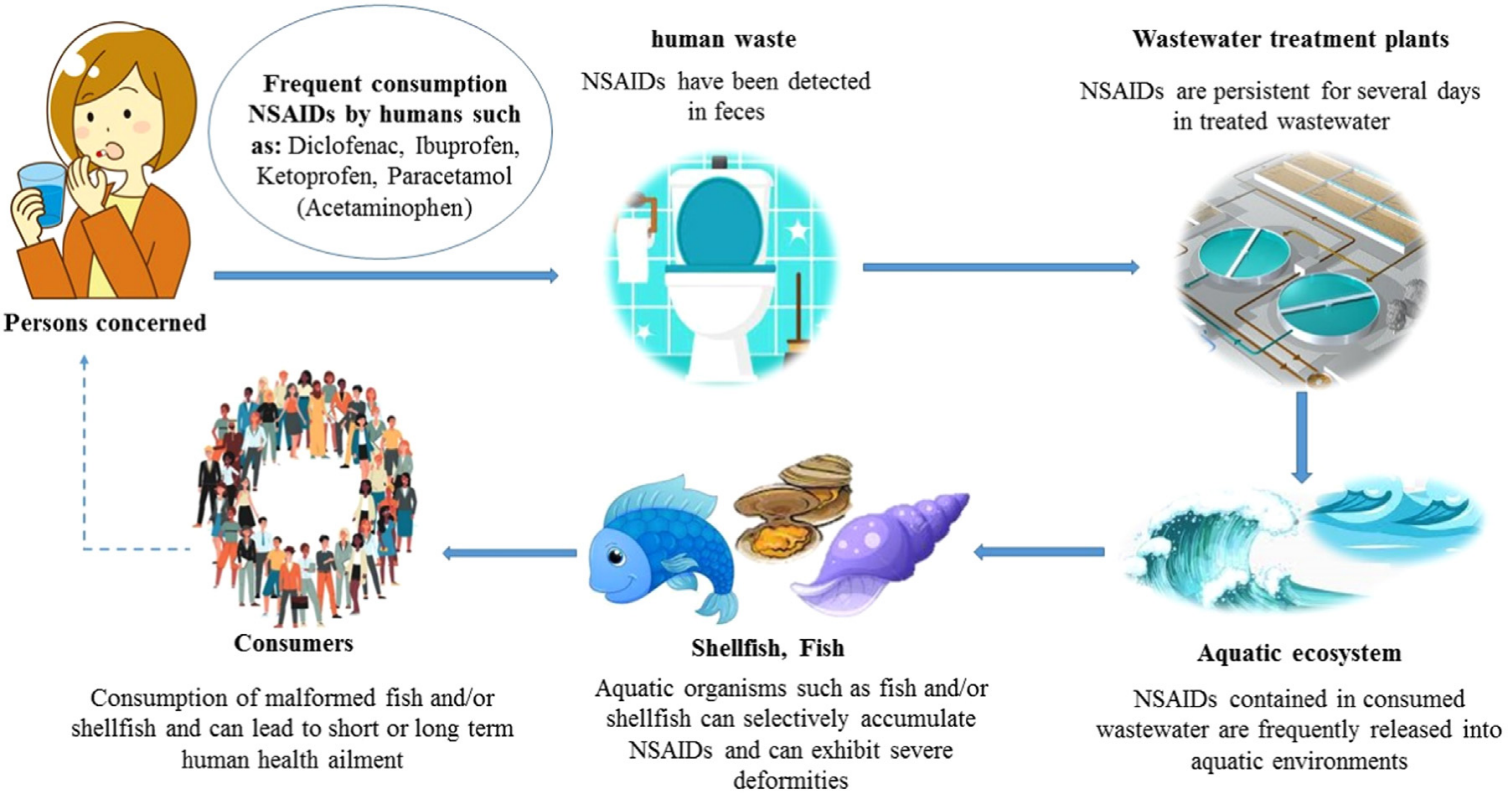
Potential foodborne transmission of non-steroidal inflammatory drugs (NSAIDs) via fish consumption by humans.

Numerous studies have shown that NSAIDs can be found in municipal wastewater in levels ranging from a few nanograms to several micrograms per liter [11]. These substances come from the excretion of unabsorbed medications in urine and feces, flushing away of unneeded medications, and industrial wastes in sanitation systems [13]. Studies that have focused on the bioaccumulation and toxicological consequences of NSAIDs and their residues in aquatic organisms have significantly expanded the literature over the past ten years [12,19] (Fig. 1).

Due to the interplay between various environmental conditions, it is challenging to establish causal links between impacts and specific contaminants in field investigations (abiotic factors, food availability, predation pressure, etc.). Laboratory tests are frequently needed as a first step in the solution to these issues in order to reduce the disrupting agents and comprehend the interaction between organisms and contaminants independently [12]. These requirements have encouraged researchers in both the academic world and the pharmaceutical sector to employ the *Danio rerio* (zebrafish) model to evaluate the ecotoxic effects of certain pollutants.

Zebrafish have developed into a popular model system for studying numerous diseases and behaviors as well as for screening for chemical and/or medication toxicity because of the genetic similarity between them and humans. This may help with drug safety issues in human patients [20], [21], [22].

The present study aims to gather information on drug concentration levels in influents and sewage treatment plant effluents in Tunisia, which may provide a unique case study compared to other nations [11]. Tunisia, a country with limited water resources, is working very hard to recycle wastewater (i.e. for irrigation and groundwater replenishment). For the safe use of the effluents from treatment facilities, it is now crucial to be aware of the current situation, which is of special interest due to the presence of both licit and illicit drugs.

The objectives of this study were to (*i*) test the acute toxicity of commonly used NSAIDs on zebrafish embryos exposed to spiked solutions of the chosen pharmaceuticals listed in Table 1, (*ii*) focus on specific abnormality endpoints (Table S1) of early developmental stages, and (*iii*) evaluate the risk of exposing zebrafish to these drugs based on measured environmental concentrations (MECs) and predicted no-effect concentrations (PNECs). Additionally, the development of a qualitative pharmacological risk assessment (QPhRA) was a goal of the paper. The risk to the environment from exposing *Danio rerio* to pharmaceutically-laced water, both individually and as part of a multi-component mixture was calculated using the Risk Quotient (RQ) method. Overall, the ecological risk assessment of individual or combination effects of NSAIDs provides a scientific foundation for highlighting current issues within the context of the QPhRA and aids in determining the future course of research.

**Table 1:** Chemical identity of the selected NSAIDs tested in the Fish Embryo Acute Toxicity (FET) test with *Danio rerio* (Source: https://go.drugbank.com/) and exposure solutions.

NSAID	Structure	CAS number	Accession number	Weight (g/mol)	Solubility in water (mg/L)	Log Kow (ECOSAR V2.2)	pKa	Nominal concentration	
								µM	mg/L
**Diclofenac**		15,307–86–5	DB00586	296.149	2.37 (at 25 °C)	4.0157	S.*A* = 4 S.*B* = −2.1	4.22 8.44 16.88 25.33 37.99	1.25 2.5 5 7.5 11.25
**Ibuprofen**		15,687–27–1	DB01050	206.2808	0.0684	3.7931	S.*A* = 4.85 S.*B* = n*.*a.	6.06 12.12 24.24 36.36 54.54	1.25 2.5 5 7.5 11.25
**Ketoprofen**		22,071–15–4	DB01009	254.2806	0.0213	3.0001	S.*A* = 3.88 S.*B* = −7.5	4.92 9.83 19.66 27.53 44.24	1.25 2.5 5 7.5 11.25
**Paracetamol (Acetaminophen)**		103–90–2	DB00316	151.1626	4.15	0.2685	S.*A* = 9.46 S.*B* = −4.4	8.27 16.54 33.08 49.62 74.42	1.25 2.5 5 7.5 11.25

n.a. = not available; S.*A* = Strongest Acidic; S.*B* = Strongest Basic.

## Method details

### Zebrafish maintenance, embryos collection and exposure

*Danio rerio*, a tropical freshwater fish, was used as a test species in the Laboratory of Molecular and Cellular Screening Processes at the Center of Biotechnology of Sfax (CBS, Tunisia). A zebrafish spawning stock, in 15 L circulation tanks, each consisting of 10 males or females separately, aged 6 to 24 months, was used for egg production. For keeping and zebrafish breeding, artificial water (AW) as specified in ISO7346–1 and 7346–2 (ISO 1996; 294.0 mg/L CaCl_2_ 2 H_2_O; 123.3 mg/L MgSO_4_ 7 H_2_O; 63.0 mg/L NaHCO_3_; 5.5 mg/L KCl) with ≥ 60% oxygen saturation and pH (7.75 ± 0.02) was used.

Each breeding group was maintained at 26 ± 1 °C with continuous aeration. A 14:10 hour photoperiod cycle (light: dark) was maintained. Adult fish were fed twice daily with commercially available dry food (e.g., TetraMin™ flakes; Tetra, Melle, Germany), occasionally, they were fed with *Artemia* *nauplius*. Breeding was achieved in chambers Aquatic Habitats (407–886–7575 made in USA) of 1.7 L of volume where males and females of zebrafish (in a ratio of 2:1) were placed with at least five replicates of rearing chambers. Rising took place within 30 min from the start of the morning light. The eggs were collected after a period of one hour of natural mating. Then the eggs were washed thoroughly and rinsed several times with AW. Healthy and developing, undamaged (injured or deformed) embryos were selected in 1 hpf for exposure testing using a Stemi 1000 /2000/2000-C stereomicroscope (Zeiss, Göttingen, Germany), transferred to crystallizing dishes and briefly stored in an incubator (26 ± 1 °C) until exposure.

A 120-hour exposure of developing zebrafish embryos, based on the OECD Guideline for Testing of Chemicals 236 - Acute Fish Embryo Toxicity (FET) test [23] under semi-static assay conditions, was performed to assess the acute toxicity of diclofenac, ibuprofen, ketoprofen and paracetamol at a common tested range of concentrations: 0; 1.25; 2.5; 5; 7.5 and 11.25 mg/L. Based on literature data, the experimental concentrations of the selected substances were chosen to span a wide range of concentrations, including relevant pharmacological and environmental concentrations [24,25].

To monitor fertilization success, freshly fertilized eggs (1 hpf) were inoculated into small petri dishes filled with the respective test solution in the first step (initiation of normal cell division and fertilization rate). During all experiments, the exposure media were changed once a day.

After verifying the success of the fertilization, the eggs were transferred individually to 24-well plates (Orange Scientific) with 2 mL of test solution per embryo. For each of the four NAIDs, five plates were prepared for each of the five concentrations (each concentration represents a plate), with twenty wells (for twenty embryos) in each plate containing the same exposure concentration but the other four wells containing 2 ml of AW and serving as a control. Each experiment was repeated three times for each of the four NAIDs. The embryos were incubated at a temperature of 26.0 ± 1.0 °C in a MODELANO 639/70 incubator under a 10:14 h dark:light regime. Every 24 h, the exposure solutions were renewed (semi-static exposure) and the lethal and sub-lethal parameters were documented (mortality, hatching and anomalies/malformations) at 24, 48, 72, 96 and 120 hpf until the end of the test. All the tests were performed in triplicate.

### Chemicals and analytical methods for exposure solution stability

The following chemicals purchased from Sigma-Aldrich (Deisenhofen, Germany) were used for the exposure of zebrafish embryos: Diclofenac (99.0 to 101.0% dried substance (ds)), ibuprofen (98.5 to 101.0% ds), ketoprofen (99.0 to 100.5% ds) and Paracetamol (99.0 to 101.0% ds). CAS numbers and other characteristics of the selected drugs are shown in Table 1 (http://www.drugbank.com). All chemicals were dissolved in AW without any auxiliary solvent.

During all experiments, the exposure media were changed once a day. For each concentration, 3 samples before (0 h) and 3 samples after exposure of 24, 48, 72 and 96 h, were collected prior the renewal of the exposure solution. In order to pinpoint any possible changes of the nominal concentration, the stability of the drugs in the exposure solutions was monitored during the four days experiment using a JENWAY 7315 spectrophotometer with 1-cm cuvette path length slot in the wavelength range of 200 – 350 nm. All stability tests were performed during 5 days and using the highest concentration of each NSAID (11.25 mg/L). After 24 of exposure the solution was renewed and the exposure was continued for 24 h more. This operation was repeated five times.

### Data analysis and morphological effect scoring

LC, EC, NOEC and LOEC, graphs and statistical analyses were calculated, designed and performed using Graphpad PrismPad 9.0.0 and 9.0.1 (151). All the driving tests were bilateral with a level of meaning at *p* < 0.05. All observations made according to OECD test guideline TG 236 (OECD 2013), were considered as a cumulative data point. According to the FET directive and as shown in Table 2, the calculation of LC is related to the presence of one of the lethal effects such as coagulation, the absence of somite formation, the heart rate or tail detachment. The EC is expressed as the cumulative effect of all sub-methods, including the major lethal parameters of the oven. Separate graphs were generated using the same PrismPad software for all observed lethal and sub-lethal effects and based on the values NOEC, LOEC and ECx. Specific effects were noted for this purpose, and the concentration-effect results were used to calculate EC_10_ values (defined as the concentration at which a 10% increase in the incidence of a monitored effect on controls could be recorded). The EC_10_ values for each compound's specific endpoints were then used to rank the compounds based on their ability to induce lethal and sub-lethal effects [26]. Morphological alterations in *Danio rerio* were documented using images captured with the ZEISS Axicocam 105 color camera and recorded on a ZEISS TV 2/3″C 0,63 × 1069–414 microscope.

**Table 2:** Cumulative lethal (LC) and sub-lethal effect concentrations (EC) as well as no observed effect concentrations (NOECs) and lowest observed effect concentrations (LOECs) after 120 hpf of exposure to Diclofenac, Ibuprofen, Ketoprofen and Paracetamol (Acetaminophen) derived from all lethal and sub-lethal endpoints in *Danio rerio* embryos.

NSAIDs	NOEC (mg/L)	LOEC (mg/L)	Sub-lethal effects (mg/L)			Lethal effects (mg/L)			ECOSAR V2.2 Fish (*Danio rerio*) LC_50_–96 hpf (mg/L)
			EC_10_	EC_20_	EC_50_	LC_10_	LC_20_	LC_50_	
Diclofenac	< 1.25	1.25	1.21	1.23	1.24	1.27	1.73	2.93	37.7
Ibuprofen	< 1.25	1.25	0.56	0.8	1.49	6.47	8.21	N.D	41.6
Ketoprofen	< 1.25	1.25	0.42	0.73	1.91	0.76	0.98	1.52	264
Paracetamol (Acetaminophen)	< 1.25	1.25	1.1	1.11	1.12	1.1	1.65	3.27	4.46E+3

N.D: Not Determined.

### Qualitative pharmaceuticals risk assessment

Anti-inflammatory drugs were the dominant therapeutic groups detected in aquatic environments [11]. In order to assess the ecological risk linked to the presence of pharmaceuticals compounds in aquatic environment based upon ecological effects data and concentration of each NSAID tested in the aquatic environment following the guidelines EC, [27] and according to the literature [15]. RQ method was applied as a novel approach to estimate the environmental risk of pharmaceuticals that are most frequently detected in wastewater effluents, surface waters and sediments [28].

The RQ for each NSAID was calculated as the ratio MEC/PNEC (Eq. (1)). MECs (mg/L), measured concentrations in realistic environment, were searched for in the scientific literature using the terms “NSAIDs”, “environment”, and “detection”, collated and percentage-ranked following [11] and listed in Table S2. The PNECs correspond to the lowest measured acute EC_50_ values for zebrafish embryos, divided by an assessment factor (AF) of 1000 (Eq. (2)). For the mixture of the four NSAIDs, RQmix was calculated by summing the individual RQ corresponding to each pharmaceutical compound [29] (Eq. (3)).

(1) RQ=MEC/PNEC

(2) PNEC=EC50/AF

(3) $$RQmix=\sum_{i=1}^{n}\left(\text{MECi}/\text{PNECi}\right)$$

Where (n) is the number of compounds considered in the mixture and (i) is the pharmaceutical compound.

Similar to RQ, the toxicity of a mixture is assessed by concentration addition (CA) model and neglected the toxic modes of action of the mixture constituents. The CA model implies that the contribution of the individual toxicants to the overall effect can be added in the form of toxic units (TU). The CA model implies that the contribution of individual toxic substances to the overall effect can be summed as TU. The TU of a mixture can be calculated following [30] (Eq. (4))

(4) $$TU=\sum_{i=1}^{n}\left(\text{MECi}/\text{EC}50\right)$$

The EC_50_ and LC_50_ values predicted by the US-EPA ECOSAR Class Program V2.2 were used to calculate the RQs, RQmix, and TUs of NSAIDs, as reported by Ben Chabchoubi et al. [31]. Four risk levels were classified using RQ and RQmix: 0.01 ≤ RQ (RQmix) < 00.1, indicate insignificant risk; 00.1 ≤ RQ (RQmix) < 0.1, indicate low risk; 0.1 ≤ RQ (RQmix) < 1, indicate moderate risk; and RQ (or RQmix) ≥ 1 indicate high ecological risk to aquatic organisms [32]. At the threshold, there is only one TU; if its value is greater than one, this indicates a potential risk [30].

## Method validation

### Chemical analysis and exposure solution stability

**Fig. 2:**
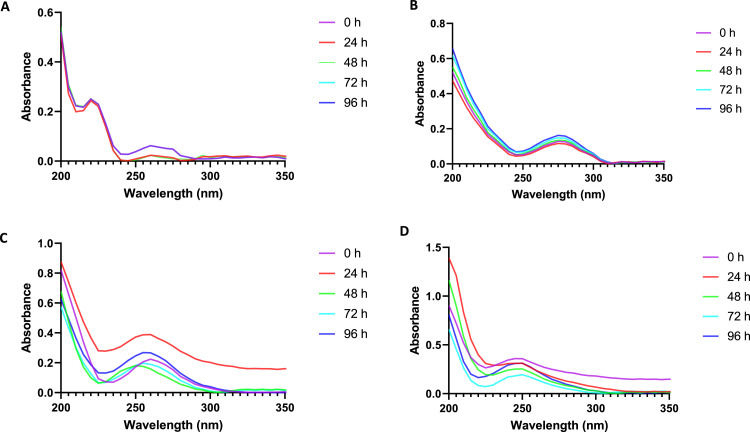
Absorbance detection of exposure solutions of Ibuprofen (A), Diclofenac (B), Ketoprofen (C) and Paracetamol (D) for recording drug peaks at the start of incubation (0 h) and at 24 h intervals (24, 48,72 and 96 h) with samples before and after exposures.

Fig. 2 and the Table S3 both display the stability of the four tested NSAIDs in the exposure solutions. Every 24 h before changing the exposure solution, the actual concentrations of the exposure medium were evaluated by an absorption measurement in order to detect any potential concentration changes. Few publications address the stability or preservation of pharmaceutical compounds during sample collection and storage from experimental or natural waters. The pharmaceutical' maximum absorbencies were measured at 275 nm for diclofenac, 225 nm for ibuprofen, 260 nm for ketoprofen, and 250 nm for paracetamol. For the purpose of determining the linearity and scope of the analytical method, the calibration graph of concentration vs. absorbance was plotted.

According to the study's findings, the four NSAIDs are stable in exposure solutions for at least 96 h. Diclofenac, ibuprofen, ketoprofen, and paracetamol have recovery rate values of 103.57, 115.22, 94.33, and 107.18, respectively.

The experimental results revealed that the NSAIDs under study, as specified in the American Standard Guide ASTM D4841, obtained between 80% and 120% of the recovery [33]. Ibuprofen and diclofenac recovery exposure concentrations over two days were marginally lower than the nominal amounts, according to Zhang et al. [34]. The actual value of exposure medium concentrations between 60 and 72 hpf was confirmed by Xia et al. [35]. The difference between the observed concentrations and the nominal amounts for ibuprofen, diclofenac, and paracetamol was within 17% for all exposure groups. According to Van den Brandhof and Montforts [36], diclofenac is stable for 72 h at all exposure concentrations. Therefore, based on the recovery trial (Table S3), the concentrations remained largely consistent during the first 96 h, which is adequate for assessing the effects of a brief NSAID exposure to *Dario rerio* and correct for its intended purpose. In general, the four chemicals' observed concentrations varied little from their nominal amounts. However, the concentrations remained constant throughout exposure, suggesting that the drugs' physical and chemical characteristics were stable in the exposure system. Additionally, recent investigations by Xia et al. [35], Van den Brandhof and Montforts [36], and Zhang et al. [34].

### NSAID-Induced general toxicity in zebrafish embryos

**Fig. 3:**
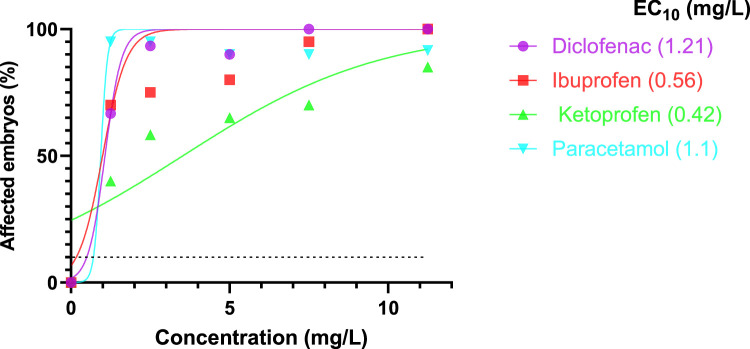
Percentage of *Danio rerio* embryos (*n* = 20 per replicate, 3 replicates) for a lethal or sub-lethal effect after 120 hpf exposure to NSAIDs. EC_10_: Effective concentration (mg/L), where 10% of embryos were affected.

An LC_50_ or an EC_50_ value is a numerical representation of a lethal or sub-lethal effect. The interest in using EC_10_ values to rule out any relevant toxicity caused by the tested pharmaceutical is growing [23]. The four test substances—diclofenac, ibuprofen, ketoprofen, and paracetamol—are arranged according to their EC_10_ values in Fig. 3. The cumulative LC and EC from all lethal and non-lethal endpoints in *Danio rerio* embryos, as well as the EC_10_ values, indicate that the toxicity of the four pharmaceuticals is as follows: ketoprofen > ibuprofen > paracetamol > diclofenac (Table 2).

The LC_50_ and EC_50_ toxicity values for ketoprofen were 1.52 and 1.91 mg/L, respectively. ketoprofen may be dangerous to aquatic and terrestrial species that are not its intended targets [37]. Ketoprofen should not be utilized in Asia for the treatment of animals, according to Naidoo et al. [38]. Ibuprofen exposure in the actual investigation did not result in an LC_50_, but rather an LC_10_ = 6.47 mg/L with the existence of malformations at an EC_50_ = 1.49 mg/L. Algae and Daphnia magna were more sensitive to the effects of ibuprofen than *Vibrio fischeri*, according to Pusceddu et al. [39] and Madikizela and Ncube [40]. Ibuprofen appears to be the product with the highest ecotoxicological risk among the NSAIDs.

It is important to note that the observed differences in computed endpoint parameters for the same drug are most likely due to the developmental stage, operating mode, and exposure circumstances [41].

In this investigation, deformities were present at an EC_50_ of 1.12 mg/L after exposure to paracetamol with an LC_50_ of 3.27 mg/L. The toxicity of paracetamol was investigated in various aquatic invertebrate species with various metabolic [42], [43], [44], physiological [45], and biochemical endpoints [46]. Deformities were found in this study at an EC_50_ of 1.12 mg/L after exposure to paracetamol with an LC_50_ of 3.27 mg/L. Paracetamol toxicity was studied in a variety of aquatic invertebrate species using a variety of metabolic [42], [43], [44], physiological [45], and biochemical endpoints [46]. Also, the exposure to Paracetamol is fatal for the majority of species such as *Danio rerio, Daphnia magna, Daphnia longispina, Vibrio fischeri, Raphidocelis subcapitata, Cylindrospermopsis raciborskii* as demonstrated by Nunes [45] and Parolini [46]. Furthermore, NAIDs have been shown to accumulate in aquatic organisms such as fish after short-term exposure with low bioconcentration or long-term exposure with high bioconcentration [47].

The LC_50_ and EC_50_ values for diclofenac toxicity at 120 hpf in this study were 2.93 mg/L and 1.24 mg/L, respectively. Diclofenac caused the death of zebrafish larvae after 48 h exposure (48 hpf-LC_50_ = 14.15 ± 0.99 mg/L) according to Zhou et al. [48]. Diclofenac also killed zebrafish larvae after 96 h exposure (96 hpf-LC_50_ = 11.25 mg/L) according to Yadav et al. [49]. Variations in mortality rates (LC_50_) can be attributed to differences in experimental environments, product cleanliness, exposure period, and zebrafish strain susceptibility [50,51].

### The FET standard sub-lethal toxicity parameters scoring

**Fig. 4:**
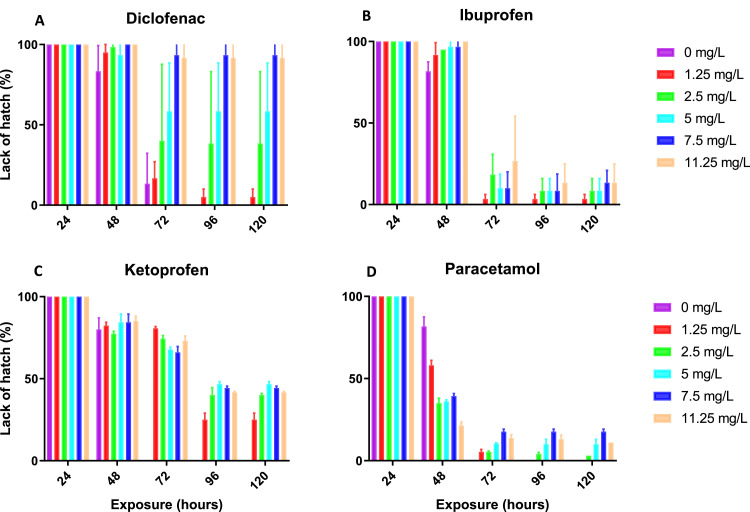
(A) Ratio of non-hatched eggs exposed to Diclofenac at different concentrations (Test Two-Way Anova with Interaction = 3.39 (*p* value = 0.0001, ***), Row Factor = 34.64 (*p* value < 0.0001, ****) and Column Factor = 24.17 (*p* value < 0.0001, ****)). (B) Ratio of non-hatched eggs exposed to Ibuprofen at different concentrations (Test Two-Way Anova with Interaction = 0.739 (*p* value = 0.7699), Row Factor = 649.8 (*p* value < 0.0001, ****) and Column Factor = 5.742 (*p* value = 0.0002, ***)). (C) Ratio of non-hatched eggs exposed to Ketoprofen at different concentrations (Test Two-Way Anova with Interaction = 12.75 (*p* value < 0.0001, ****), Row Factor = 73.10 (*p* value < 0.0001, ****) and Column Factor = 13.71 (*p* value < 0.0001, ****)). (D) Ratio of non-hatched eggs exposed to Paracetamol at different concentrations (Test Two-Way Anova with Interaction = 6.48 (*p* value = 0.0001, ****), Row Factor = 92.75 (*p* value < 0.0001, ****) and Column Factor = 0.618 (*p* value < 0.0001, ****)).

**Fig. 5:**
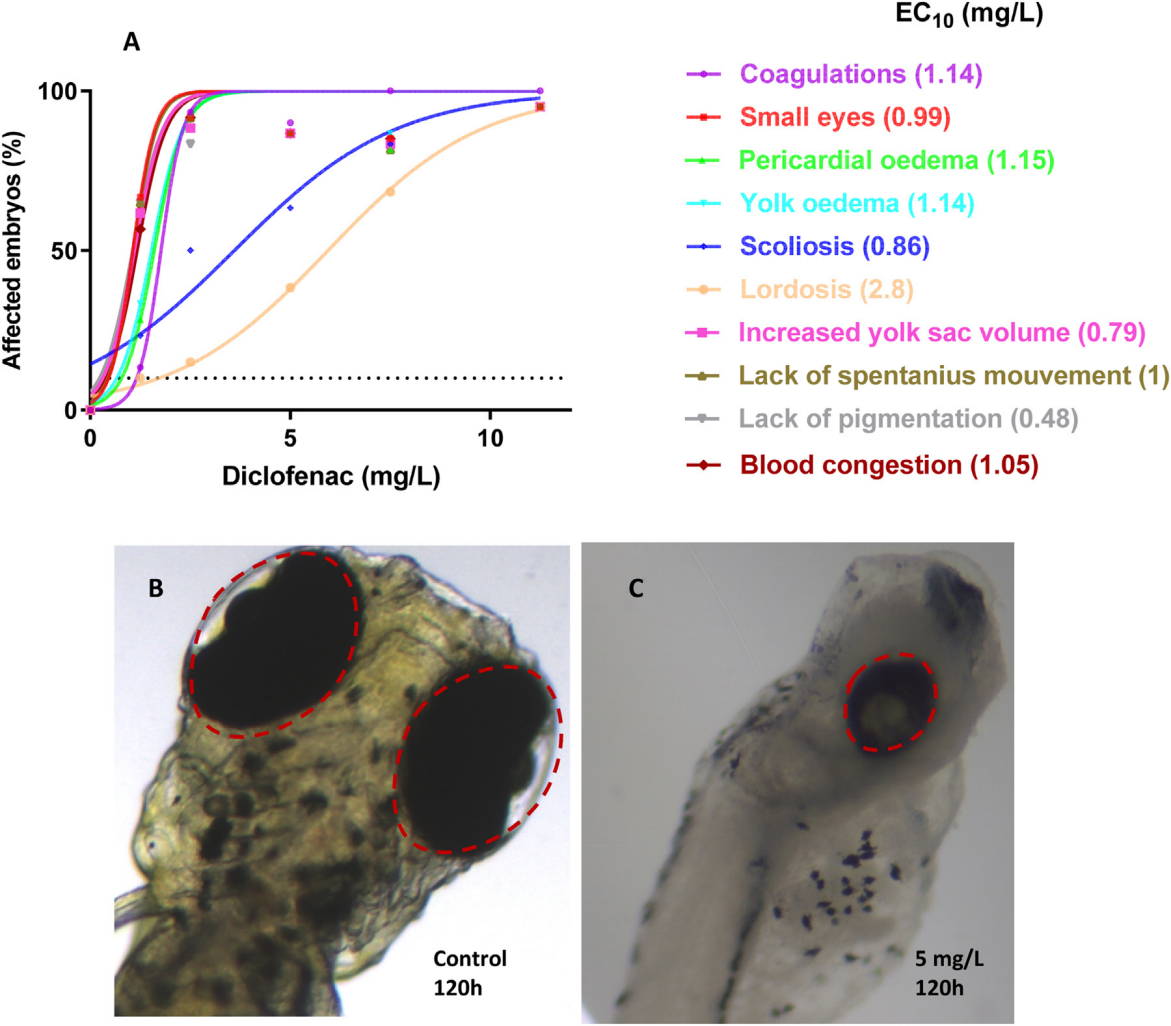
(A) Specific sub-lethal effects recorded in zebrafish (*Danio rerio*) embryos exposed for 120 hpf to Diclofenac; The data are given in% of affected embryos (*n* = 20 per replicate, 3 replicates); (B) Normal eye development in 120 hpf zebrafish embryos; (C) "Small eyes" endpoint in 120 hpf zebrafish embryos after treatment with 5 mg/L diclofenac; Area of interest framed in red.

First, the effects of four NSAIDs exposure on zebrafish egg hatch rate and embryo development were evaluated (Fig. 4). The incidences of unhatched eggs for the substances with the greatest exposure concentrations were 90, 16.55, 42, and 11%, respectively, after 120 h of exposure to diclofenac, ibuprofen, ketoprofen, and paracetamol. At the end of the experiment, unhatched eggs are dead. Observed effects of each tested drug on larvae, are detailed in the Fig. 5 as a function of their EC_10_. The obtained values of ECx, LOEC and NOEC are shown in Table S4.

EC_10_ values were determined for certain effects, but not for others due to software constraints and the limited occurrence of 10% effects at the highest test concentration (only). According to their EC_10_ values, the compounds were sorted for these particular effects as follow:

• Diclofenac has more severe toxicity effect than ibuprofen for inducing scoliosis and yellow edema;
• Diclofenac decreases significantly the yolk sac volume, reduces the eye size, and leads to stronger blood congestion than ibuprofen and ketoprofen;
• Diclofenac is more toxic than ibuprofen and ketoprofen for causing depigmentation of the skin, pericardial edema, and lack of spontaneous movements;

This grouping revealed that diclofenac, followed by ibuprofen and/or ketoprofen, is more hazardous for the various abnormalities seen.

Particular observed effects on exposed zebrafish embryos to the four NSAIDs are listed for each chemical in Table 3 and were divided into common and uncommon categories using the OECD (2013) criteria. According to the set of common endpoints, blood congestion and small eyes are the two most often found abnormalities following exposure to the four NAIDs. As seen in Table 3, specific comments have been provided in circumstances where a clear categorization could not be determined, including *, **, ***, and ****. Ibuprofen, ketoprofen, and diclofenac all cause the same abnormalities in the larvae but with different intensities.

**Table 3:** Selected observed effects in zebrafish embryos for Diclofenac, Ibuprofen, Ketoprofen and Paracetamol, split into OECD 236 as common and uncommon endpoints.

Malformations		NSAIDs			
		Diclofenac	Ibuprofen	Ketoprofen	Paracetamol (Acetaminophen)
**OECD TG 236 endpoints**	Coagulations	I: ⁎⁎⁎⁎	I: ⁎⁎	I: ⁎⁎	I: *
	Lack of somite formation	–	–	–	–
	Tall detached	–	–	–	–
	Missing heartbeat	E: ⁎⁎	–	–	–
**Selected common endpoints**	Blood congestion	L: ⁎⁎⁎⁎	L: ⁎⁎⁎⁎	N: *	O: *
	Small eyes	L: ⁎⁎⁎⁎	L: ⁎⁎⁎⁎	L: ⁎⁎⁎	L: ⁎⁎⁎⁎
**Selected uncommon endpoints**	Increased yolk sac volume	I: ⁎⁎⁎⁎	L: ⁎⁎⁎	L: ⁎⁎	–
	Lack of pigmentation	L: ⁎⁎⁎⁎	L: ⁎⁎⁎⁎	L: ⁎⁎⁎	–
	Scoliosis	L: ⁎⁎⁎⁎	L: ⁎⁎⁎	L: *	–
	Lordosis	L: ⁎⁎⁎⁎	O: *	N: *	–
	Lack of spontaneous movement	L: ⁎⁎⁎⁎	N: ⁎⁎	L: ⁎⁎⁎	–
	Pericardial edema	L: ⁎⁎⁎⁎	L: ⁎⁎⁎⁎	N: ⁎⁎⁎	–
	Yolk edema	L: ⁎⁎⁎⁎	L: ⁎⁎⁎⁎	L: ⁎⁎	–

Number of malformations appearing per replicate:.

⁎ number of malformations appeared < 5.

⁎⁎ 5 < number of malformations appeared < 10.

⁎⁎⁎ 10 < number of malformations appeared < 15.

⁎⁎⁎⁎ 15 < number of malformations appeared = 20 hpf: hours post fertilization A: 24 hpf; B: 48 hpf; C: 72 hpf; D: 96 hpf; E: 120 hpf; F: 24 – 48 hpf; G: 24 – 72 hpf; H: 24 – 96 hpf; I: 24 – 120 hpf; J: 48 – 72 hpf; K: 48 – 96 hpf; L: 48 – 120 hpf; M: 72 – 96 hpf; N: 72 – 120 hpf; O: 96 – 120 hpf.

In the case of diclofenac, no heartbeat was seen for a number of larvae less than 50% after 120 h of exposure, confirming their mortality, in accordance with OECD (2013) endpoints. Among all potential malformations, eye constriction was the most delicate one displayed in Fig. 5 after diclofenac exposure. Indeed, embryos exposed to diclofenac showed an increase in the number of individuals with reduced eye size.

**Fig. 6:**
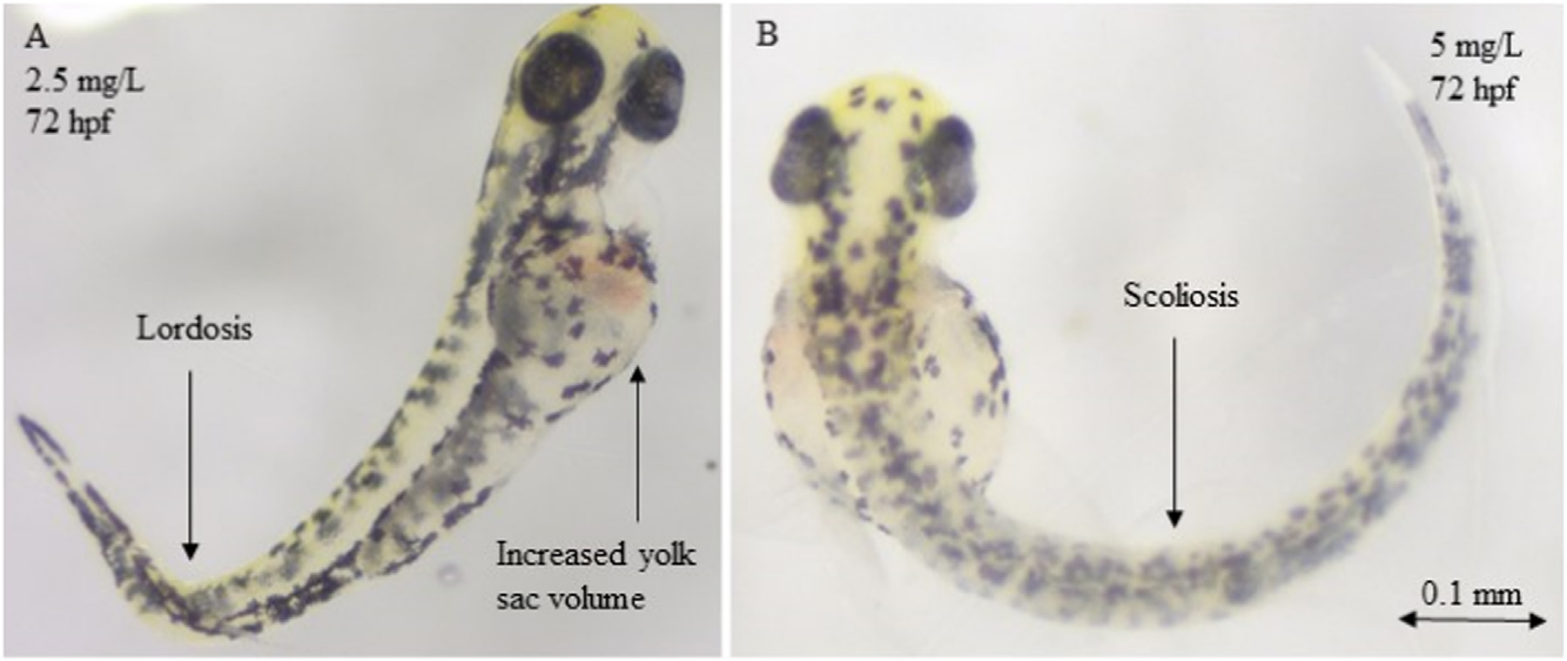
(A) Lordosis, Increased yolk sac volume (Diclofenac 2.5 mg/L, 72 hpf) and (B) Scoliosis (Diclofenac 5 mg/L, 72 hpf) are three of the most prominent observations after exposure of zebrafish embryos to diclofenac and other NSAIDs.

The data and graphs for the other three NSAIDs are provided in Table S4 and Fig S1. With most NSAIDs, scoliosis and lordosis may be observed as frequent (non-specific) side effects, and they may be considered the most important findings (Fig. 6). The most notable findings following exposure to diclofenac included scoliosis and lordosis deformities, which are listed as rare consequences with specific EC_10_ data. Diclofenac's EC_10_ for developing scoliosis was determined to be 0.86 mg/L, but its EC_10_ for inducing lordosis is estimated to be 2.8 mg/L (Fig. 6). The EC_10_ data for scoliosis and lordosis in the case of the ketoprofen and ibuprofen is presented in Table S4 and Fig S1. In the case of paracetamol, these abnormalities were absent. However, a thorough effects analysis also revealed that the most sensitive endpoints for all substances—aside from paracetamol—were the shrinkage of the eyes (Fig. 5) and the skin depigmentation.

Lethal and sublethal effects analyses, along with the search for surrogate endpoints in zebrafish embryos for risk assessment of NSAIDs by zebrafish embryos, generally increase the sensitivity and predictability of FET for toxicity screening developmental. The present investigation was specifically guided by the following two key questions: (1) When compared to a typical study that combines all observations into a single summary parameter (as required by the OECD 2013 guideline), would an independent assessment of all impacts improve the zebrafish embryo's prediction ability for mammalian and human toxicity and teratogenicity? Which NSAIDs would likewise be teratogenic in the zebrafish embryo, predicting the zebrafish embryo would anticipate known in vivo-negative and/or in vivo-positive potentials of NSAIDs?

Regarding the first methodological concern, it was found that when the isolated analysis was compared to the summary analysis of the parameters, the findings drawn from the two methodologies with regard to the predictability of the effects in vertebrates were similar. However, the isolated strategy enables a more direct comparison of FET and vertebrate data. The frequency of malformations observed for the 4 NSAIDs could be directly compared, but a summary analysis of all the effects followed by an alignment according to their EC_10_ values only allowed for the identification of a trend toward increased or decreased toxicity. Additionally, a similar order to that discovered for general effects was obtained when sorting compounds for these particular effects by EC_10_ values.

Ranking the four tested drugs according to their EC_10_ values, the findings of the summary analysis for the second issue (prediction of teratogenic potentials) showed that ibuprofen and ketoprofen were the most dangerous substances for fish embryos (Fig. 3 and Table 2). Consequently, the zebrafish embryo is rated as a “good” alternative toxicity test by the European Center for the Validation of Alternative Methods (ECVAM) assessment standards [52,53].

### Cardiac developmental toxicity

**Fig. 7:**
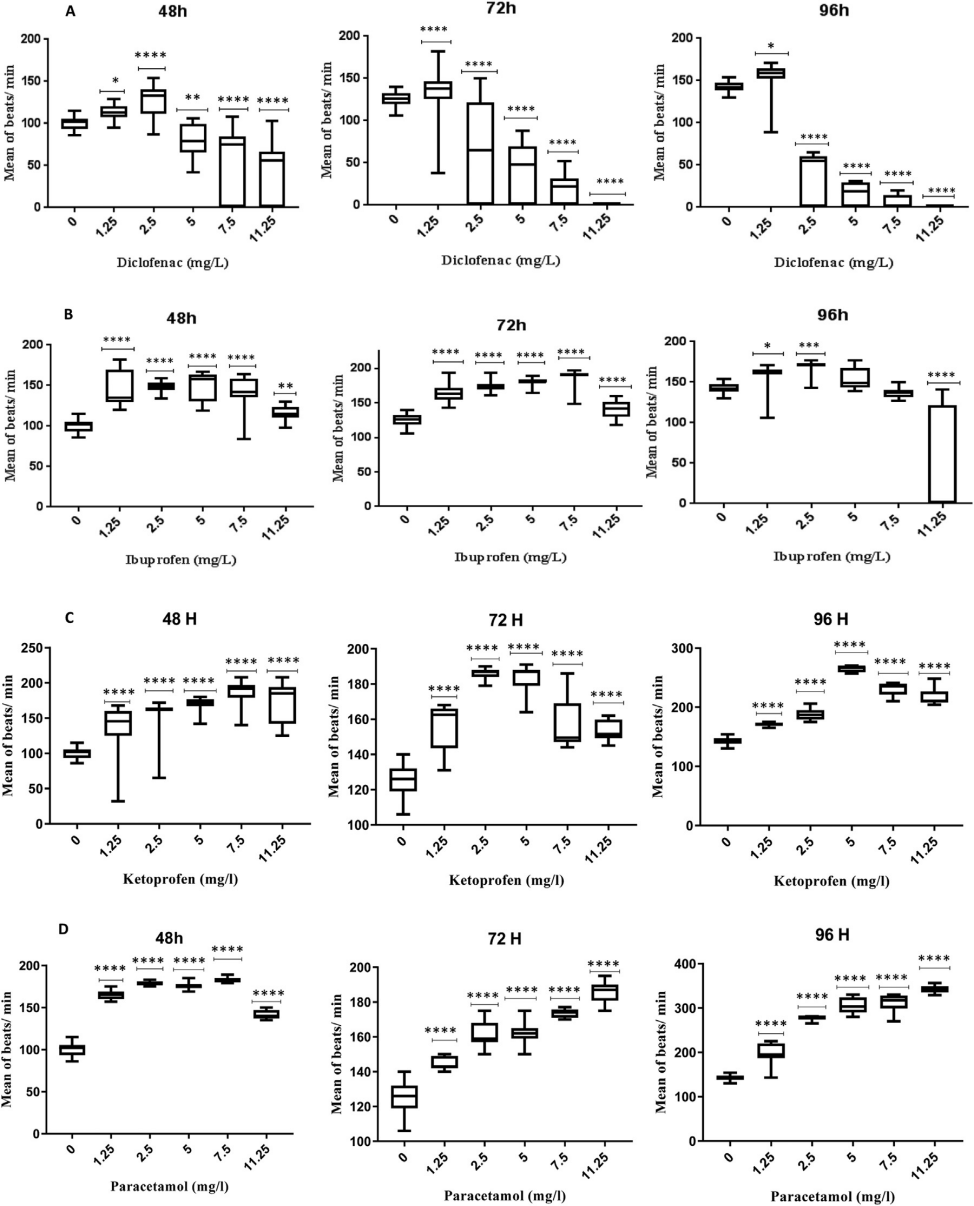
Heartbeat rate of zebrafish larvae recorded after 48, 72 and 96 hpf in response to Diclofenac (A), Ibuprofen (B), Ketoprofen (C) and Paracetamol (D) exposure; Ordinary one-way ANOVAs with multiple comparisons test were applied; *: *p* value < 0.05; **: *p* value < 0.01; ***: *p* value < 0.001; ****: *p* value < 0.0001.

Toxic levels of zebrafish were measured using heart rates and pericardial edema. The greatest deformative anomaly found while examining the morphology of zebrafish embryos after exposure to ibuprofen, ketoprofen, diclofenac, and paracetamol was pericardial edema. The Fig. 7 depicts the results of a 60 second heartbeat test on 10 randomly chosen larvae at 48, 72, and 96 hpf for each replicate. The heart rate was significantly elevated by diclofenac at doses of 1.25 and 2.5 mg/L, peaking at 127.5 beats per minute at 2.5 mg/L, as shown in Fig. 7A.

The heartbeats dramatically dropped for doses of 5, 7.5, and 11.25 mg/L, and at 11.25 mg/L they reached a minimum rate of 42.04 beats per minute. At 72 and 96 hpf, however, the heartbeats continued to occur at the same pace, reaching a maximum at 1.25 mg/L and then falling again from 2.5 to 11.25 mg/L, when they reached their minimum rates of 19.27 and 0 beats per minute for 72 and 96 hpf, respectively.

For ibuprofen, both 48 and 72 hpf had the same cardiac tracking diagram pace (Fig. 7B). For all concentrations, the heartbeat was noticeably faster than the control. With 160.5 and 170.2 beats per minute for 96 hpf, the heart rate increased by 1.25 and 2.5 mg/L, respectively. The Fig. 7B also points out that although there was no discernible difference at doses of 5 and 7.5 mg/L, a considerable drop in heart rate—56.97 beats per minute—was detected at 11.25 mg/L. Ketoprofen significantly increased heart rate at all concentrations at 48, 72, and 96 hpf, peaking at 5 mg/L with 265.4 beats per minute at the latter time point (Fig. 7C). For all concentrations of paracetamol, the pulse rate per minute significantly increased at 48, 72, and 96 hpf (Fig. 7D), which explains the hyperactivity of larvae seen in the aquarium.

The embryos' delayed development of their hearts during their early development is correlated with changes in cardiac output [34]. After hatching or even when the zebrafish embryo's circulatory system is already well-developed, specific cardiovascular toxicity may manifest. This dysfunction was also consistent with the development of zebrafish cardiac abnormalities, such as pericardial edema (EC_50_ Diclofenac = 1.3 mg/L; EC_50_ Ibuprofen = 9.18 mg/L). This is not to discount the effects of ibuprofen and diclofenac on the cardiovascular system of zebrafish. These results concur with those of Zhang et al. [34], who carried out more cardiac and physiological research at various stages of the embryonic development. Ibuprofen and diclofenac decreased heart rate in embryos during early stages of development (30, 36, and 44 hpf), but after 56 h of exposure, the heart rates for ibuprofen and diclofenac returned to normal.

The lower heart rate in the current study would be directly associated with the inhibition of embryonic development caused by ibuprofen and diclofenac, which would result in a delay in hatching processes. The aforementioned aberrations were found to be caused by ibuprofen and diclofenac exposure, which also accounts for the fish's cardiac arrhythmia. It is widely acknowledged that using NSAIDs raises the risk of cardiovascular events like ischemic stroke, heart failure, and cardiac arrest [34,54]. This is in line with the results that have been observed in zebrafish embryos.

### Determination of risk quotient (RQ)

By calculating the individual RQs, the expected mixture (RQmix) effect and the TU values, the environmental risks of diclofenac, ibuprofen, ketoprofen, and paracetamol were estimated. The outcomes of the risk assessment are shown in Tables 4 and S5.

**Table 4:** The risk quotient (RQ), mix risk quotient (RQmix) and Toxic units (TU) qualification corresponding to the measured environmental concentration (MEC) of the tested NSAIDs.

It is important to note that in this study, RQs are calculated using two different models, the first of which is based on EC_50_ values determined in the current work (Table S4), and the second of which is predicted using the ECOSAR software. The four risk levels reported in the qualitative pharmaceuticals risk assessment section were used to interpret the findings. The fact that the majority of the results were greater than 1.0 suggests that the NSAIDs chosen pose a significant threat to the ecosystem.

Based on the concentrations of pharmaceutical products found in the aquatic habitats of Tunis, France, Kenya, Nigeria, Portugal, and Minnesota, we calculated the RQs and RQmix of the four pharmaceutical compounds. Fish are still exposed to a variety of medicinal mixtures in bodies of water. A combined risk assessment based on data on acute single-substance fish toxicity in *Danio rerio* was used to assess the ecological risk of several pharmaceutical mixtures. The frequency of exceeding (RQmix > 1) is shown in Table 4 where in that case samples are considered of high risk and should be treated with caution. Table 4 also shows the results of TU's assessment of the combined effects of the four NSAIDs studied. The reduction in risk levels was noticeable when compared to the RQmix, despite the fact that there was only one rank order difference. The risk ranking difference between RQmix and TU reveals how vulnerable the species is during these critical early developmental stages. Thus, geographical differences between countries and activities at sampling regions and locations can result in significant differences in RQ, RQmix, and TU results.

Conventional risk assessment methods used in RQ, RQmix, and TU results may not take into account all effects data, such as chronic data (carcinogenic-mutagenic-reprotoxic or endocrine disruptor effect), and thus may not reach a conclusion about risk that is both comprehensive and protective for these Persistent, Bioaccumulative, and Toxic (PBT) substances. However, the majority of the thousands of NSAIDs on the market have a severe lack of information about their Persistence, Bioaccumulation, and Toxicity (PBT) [55]. As a result, it is critical in the future to base hazard assessment on chronic toxicity information, ideally supplemented with kinetic data that allows the derivation of critical body burden or internal dose information.

## Conclusion

In conclusion, all of the NSAIDs tested had a significant effect on the embryonic and larval development of zebrafish. The findings showed that diclofenac, ketoprofen, and ibuprofen had high toxicity data, whereas paracetamol had extremely low toxicity data.

The findings also showed that the NSAIDs used in this study had RQs greater than one and that appropriate toxicity data are required for an accurate assessment of the ERA.

Overall, additional risk assessment studies involving other taxa of the aquatic environment and both acute and chronic exposures for the four NAIDS should be conducted to fully understand the risk posed by pharmaceuticals on the environment.

Despite the clarity of the conclusions drawn from the scientific data published in this issue, the majority of countries are unaware of the environmental dangers of this type of NAIDS, and the pressure of pharmaceutical residues on aquatic organisms will continue to rise. As a result, strict guidelines for mitigation measures and legislative changes for pharmaceutical release into the environment have become a global priority.

## Ethics statements

All experimental procedures on animals were performed in line with the principles of the Declaration of Helsinki. Approval was granted by the Animal Ethics Committee of the National School of Veterinary Medecine of Sidi Thabet, University of Manouba, Tunisia, on the 1st February 2022 under the code number CEEA-ENMV 44/22.

## Funding

This research did not receive any specific grant from funding agencies in the public, commercial, or not-for-profit sectors.

## CRediT authorship contribution statement

**Imen Ben Chabchoubi:** Investigation, Methodology, Software, Data curation, Formal analysis, Writing – original draft, Writing – review & editing. **Rim Attya Bouchhima:** Software, Data curation. **Nacim Louhichi:** Resources, Validation. **Aissette Baanannou:** Resources, Validation. **Saber Masmoudi:** Resources. **Olfa Hentati:** Conceptualization, Methodology, Resources, Formal analysis, Supervision, Writing – review & editing, Validation.

## Declaration of Competing Interest

The authors declare that they have no known competing financial interests or personal relationships that could have appeared to influence the work reported in this paper.

## Data Availability

All data generated or analyzed during this study are included in this published article and its supplementary information files.

## Appendix Supplementary materials

This paper contains supplementary information available in Fig. S1, and in Tables S1–S5.

Image, application 1

Image, application 2

Image, application 3

Image, application 4

Image, application 1

Image, application 6
