# Supplementary material for: Short-term effects of various non-steroidal anti-inflammatory drugs (NSAIDs) on Danio rerio embryos
Source: MethodsX. 2023 May 11;10:102215. doi: 10.1016/j.mex.2023.102215 (PMC10209031; doi:10.1016/j.mex.2023.102215)
Supplement: Supplementary file 1 [file mmc1.docx]

**A**

**B**

**C**

**Fig S1**. Specific sub-lethal endpoints recorded in zebrafish (*Danio rerio*) embryos exposed for 120 hpf to Ibuprofen (**A**), Ketoprofen (**B**) and Paracetamol (**C**); The data are given in % of affected embryos (n = 20 per replicate, 3 replicates); N.D: Not Determined.
