# Supplementary material for: Short-term effects of various non-steroidal anti-inflammatory drugs (NSAIDs) on Danio rerio embryos
Source: MethodsX. 2023 May 11;10:102215. doi: 10.1016/j.mex.2023.102215 (PMC10209031; doi:10.1016/j.mex.2023.102215)
Supplement: Supplementary file 2 [file mmc2.docx]

**Table S1**. List of recorded endpoints in *Danio rerio* embryos exposed to Diclofenac, Ibuprofen, Ketoprofen and Paracetamol (Acetaminophen) at 120 hpf according Fish Embryo Acute Toxicity (FET) test

| **Core endpoints of lethality**  **(OECD TG 236; OECD 2013)** | **Sub-lethal endpoints**  **(“any other observation”)** |
| --- | --- |
| - Coagulation - Lack of heartbeat - Lack of tail detachment - Lack of somite formation | - Axial malformations (Scoliosis) - Axial malformations (Lordosis) - Blood Congestion - Lack of pigmentation - Pericardial edema - Small eyes - Spontaneous movement - Yolk edema - Increased yolk sac volume |
