# Supplementary material for: Short-term effects of various non-steroidal anti-inflammatory drugs (NSAIDs) on Danio rerio embryos
Source: MethodsX. 2023 May 11;10:102215. doi: 10.1016/j.mex.2023.102215 (PMC10209031; doi:10.1016/j.mex.2023.102215)
Supplement: Supplementary file 3 [file mmc3.docx]

**Table S2**. Measured environmental concentrations (MECs) of selected NSAIDs

| **NSAID** | **Types of water** | **Environmental concentrations (median)** | **Country** | **References** |
| --- | --- | --- | --- | --- |
| **Diclofenac** | Surface water  Ground water  Sachet water | < 1 - 200 ng/L (12 ng/L)  < 1 - 42 ng/L (< 1 ng/L)  < 1 ng/L (< 1 ng/L) | Nigeria | [16] |
|  | WWTPs | 294.00941 mg/L | France | [12] |
|  | Guadiana basin | 4806 ng/L | Portugal | [4] |
|  | Hospital wastewater  WWTP  Coastal water | n.d. - 8 ng/L  Inf: n.d. - 11 ng/L; Eff: 32 - 70 ng/L  n.d. - 23 ng/L | Tunisia | [11] |
| **Ibuprofen** | Surface water  Ground water  Sachet water | < 4 - 2,740 ng/L (298 ng/L)  < 4 - 2,250 ng/L (32 ng/L)  < 4 - 50 ng/L (12 ng/L) | Nigeria | [16] |
|  | Guadiana basin | 3161 ng/L | Portugal | [4] |
| **Ketoprofen** | WWTPs | 255.1017 mg/L | France | [12] |
|  | Guadiana basin | 321.40 ng/L | Portugal | [4] |
|  | Hospital wastewater  WWTP  Coastal water | 200 - 18,100 ng/L  Inf: 1,200 - 3,300 ng/L; Eff: 350 - 790 ng/L  n.d. - 76 ng/L | Tunisia | [11] |
| **Paracetamol (Acetaminophen)** | Surface water  Ground water  Sachet water | 1 - 12,430 ng/L (24 ng/L)  < 1 - 188 ng/L (7 ng/L)  < 1 - 11 ng/L (1 ng/L) | Nigeria | [16] |
|  | Nairobi river | 31.003 mg/L | Kenya | [5] |
|  | WWTPs | 246.1237 mg/L | France | [12] |

WWTP Inf: Wastewater treatment plant influent

WWTP Eff: Wastewater treatment plant effluent

WW from TPs: Wastewater from treatment plants

n.d.: not determined
