# Supplementary material for: Short-term effects of various non-steroidal anti-inflammatory drugs (NSAIDs) on Danio rerio embryos
Source: MethodsX. 2023 May 11;10:102215. doi: 10.1016/j.mex.2023.102215 (PMC10209031; doi:10.1016/j.mex.2023.102215)
Supplement: Supplementary file 4 [file mmc4.docx]

**Table S3. Calibration table of UV spectrophotometric method for the tested NSAIDs**

| **NSAIDs** | **Nominal concentration** | **λ (nm)** | | **Absorbance** | | | | |
| --- | --- | --- | --- | --- | --- | --- | --- | --- |
|  |  |  |  | **0h** | **24h** | **48h** | **72h** | **96h** |
| Ibuprofen | Before exposure 7.5 mg/L | 225 | 0.229 | | 0.221 | 0.228 | 0.229 | 0.229 |
| Diclofenac | Before exposure 5 mg/L | 275 | 0.128 | | 0.126 | 0.134 | 0.148 | 0.162 |
| Ketoprofen |  | 260 | 0.172 | | 0.21 | 0.159 | 0.189 | 0.172 |
| Paracetamol (Acetaminophen) |  | 250 | 0.327 | | 0.312 | 0.253 | 0.262 | 0.312 |
| **NSAIDs** | **Calibration curve of NSAIDs** | | **Concentration (mg/L)** | | | | | |
|  |  |  | **0h** | | **24h** | **48h** | **72h** | **96h** |
| Diclofenac | Y = 0.0313 X with R² = 0.9997 | | 4.099 | | 4.039 | 4.296 | 4.747 | 5.178 |
| Ibuprofen | Y = 0.0265 X with R² = 0.9999 | | 8.642 | | 8.339 | 8.604 | 8.642 | 8,642 |
| Ketoprofen | Y = 0.0365 X with R² = 0.9997 | | 4.705 | | 5.756 | 4.342 | 5.168 | 4.716 |
| Paracetamol (Acetaminophen) | Y = 0.0582 X with R² = 0.9998 | | 5.613 | | 5.352 | 4.351 | 4.518 | 5.359 |
| **NSAIDs** | **Stability parameter** | | **0h** | | **24h** | **48h** | **72h** | **96h** |
| Diclofenac | % Recovery | | 81.982 | | 80.793 | 85.912 | 94.931 | 103.573 |
| Ibuprofen |  |  | 115.220 | | 111.195 | 114.717 | 115.220 | 115.220 |
| Ketoprofen |  |  | 94.109 | | 115.091 | 86.853 | 103.363 | 94.325 |
| Paracetamol (Acetaminophen) |  |  | 112.261 | | 107.049 | 87.028 | 90.334 | 107.179 |

Where: % Recovery = (determined concentration / reference concentration) x 100
