# Supplementary material for: Short-term effects of various non-steroidal anti-inflammatory drugs (NSAIDs) on Danio rerio embryos
Source: MethodsX. 2023 May 11;10:102215. doi: 10.1016/j.mex.2023.102215 (PMC10209031; doi:10.1016/j.mex.2023.102215)
Supplement: Supplementary file 5 [file mmc5.docx]

**Table S4.** Endpoint-specific effect concentrations (EC) as well as no observed effect concentrations (NOECs) and lowest observed effect concentrations (LOECs) in *Danio rerio* embryos after 120 hpf of exposure to Diclofenac, Ibuprofen, Ketoprofen and Paracetamol (Acetaminophen)

| **Malformations** | **Drug** | **Toxicity parameters** | | | | |
| --- | --- | --- | --- | --- | --- | --- |
|  |  | **NOEC (mg/L)** | **LOEC (mg/L)** | **EC_10_ (mg/L)** | **EC_20_ (mg/L)** | **EC_50_ (mg/L)** |
| **Scoliosis** | Diclofenac | < 1.25 | 1.25 | 0.86 | 1.36 | 2.99 |
|  | Ibuprofen | 1.25 | 2.5 | 5.09 | 6.57 | 10.16 |
|  | Ketoprofen | 5 | 7.5 | - | - | - |
|  | Paracetamol (Acetaminophen) | - | - | - | - | - |
| **Lordosis** | Diclofenac | 1.25 | 2.5 | 2.8 | 4.19 | 8.32 |
|  | Ibuprofen | < 1.25 | 1.25 | 2.11 | 10.49 | N.D |
|  | Ketoprofen | - | - | - | - | - |
|  | Paracetamol (Acetaminophen) | - | - | - | - | - |
| **Blood congestion** | Diclofenac | < 1.25 | 1.25 | 1.05 | 1.11 | 1.2 |
|  | Ibuprofen | 2.5 | 5 | 5.78 | 6.6 | 8.29 |
|  | Ketoprofen | - | - | - | - | - |
|  | Paracetamol (Acetaminophen) | 7.5 | 11.25 | - | - | 11.74 |
| **Coagulation** | Diclofenac | < 1.25 | 1.25 | 1.14 | 1.36 | 1.56 |
|  | Ibuprofen | 2.5 | 5 | 6.99 | 8.47 | 11.74 |
|  | Ketoprofen | < 1.25 | 1.25 | 0.007 | 0.11 | 11.2 |
|  | Paracetamol (Acetaminophen) | 1.25 | 2.5 | N.D | N.D | N.D |
| **Lack of pigmentation** | Diclofenac | < 1.25 | 1.25 | 0.42 | 0.48 | 0.6 |
|  | Ibuprofen | < 1.25 | 1.25 | 1.5 | 2.69 | 7.21 |
|  | Ketoprofen | 2.5 | 5 | N.D | N.D | 2.03 |
|  | Paracetamol (Acetaminophen) | - | - | - | - | - |
| **Pericardial edema** | Diclofenac | < 1.25 | 1.25 | 1.15 | 1.21 | 1.3 |
|  | Ibuprofen | 2.5 | 5 | 7.22 | 7.89 | 9.18 |
|  | Ketoprofen | 5 | 7.5 | N.D | N.D | 8.48 |
|  | Paracetamol (Acetaminophen) | - | - | - | - | - |
| **Yolk edema** | Diclofenac | < 1.25 | 1.25 | 1.14 | 1.19 | 1.49 |
|  | Ibuprofen | 2.5 | 5 | 7.21 | 7.88 | 9.18 |
|  | Ketoprofen | - | - | - | - | - |
|  | Paracetamol (Acetaminophen) | - | - | - | - | - |
| **Small eyes** | Diclofenac | < 1.25 | 1.25 | 0.99 | 1.05 | 1.15 |
|  | Ibuprofen | < 1.25 | 1.25 | 0.29 | 0.65 | 2.52 |
|  | Ketoprofen | 7.5 | 11.25 | - | - | 9.16 |
|  | Paracetamol (Acetaminophen) | 7.5 | 11.25 | - | - | - |
| **Lack of spontaneous movement** | Diclofenac | < 1.25 | 1.25 | 1 | 1.06 | 1.16 |
|  | Ibuprofen | 2.5 | 5 | 5.1 | 6.89 | 11.54 |
|  | Ketoprofen | 2.5 | 5 | N.D | N.D | 2.34 |
|  | Paracetamol (Acetaminophen) | - | - | - | - | - |
| **Increased yolk sac volume** | Diclofenac | < 1.25 | 1.25 | 0.79 | 0.87 | 1.03 |
|  | Ibuprofen | 2.5 | 5 | 5.3 | 5.46 | 9.3 |
|  | Ketoprofen | 1.25 | 2.5 | 1.28 | 2.83 | 10.89 |
|  | Paracetamol (Acetaminophen) | - | - | - | - | - |

N.D: Not Determined

- : malformation not observed
