# Supplementary material for: Short-term effects of various non-steroidal anti-inflammatory drugs (NSAIDs) on Danio rerio embryos
Source: MethodsX. 2023 May 11;10:102215. doi: 10.1016/j.mex.2023.102215 (PMC10209031; doi:10.1016/j.mex.2023.102215)
Supplement: Supplementary file 6 [file mmc6.docx]

**Table S5.** The PNEC (**A**) risk quotient (RQ) (**B**), mix risk quotient (RQmix) and Toxic units (TU) (**C**) calculation corresponding to the measured environmental concentrations (MEC) of tested NSAIDs

**A**

| PhACs | The present study | | ECOSAR V2.2 | |
| --- | --- | --- | --- | --- |
|  | **EC_50_** | **PNEC = EC_50_/AF** | **EC_50_** | **PNEC = EC_50_/AF** |
| Diclofenac | 1.24 | 0.00124 | 37.7 | 0.0377 |
| Ibuprofen | 1.49 | 0.00149 | 41.6 | 0.0416 |
| Ketoprofen | 1.91 | 0.00191 | 264 | 0.264 |
| Paracetamol (Acetaminophen) | 1.12 | 0.00112 | 4.46E+03 | 4.46 |

Where AF (Assessment Factor) = 1000

**B**

| PhACs | Types of waters | | | MEC (mg/L) | The present study | | ECOSAR V2.2 | | Sampling location | References |
| --- | --- | --- | --- | --- | --- | --- | --- | --- | --- | --- |
|  |  |  |  |  | **RQ** | **MEC/EC_50_** | **RQ** | **MEC/EC_50_** |  |  |
| Diclofenac | Surface water | | | 0.2 | 161.290 | 0.16129 | 5.3050 | 0.005305 | Nigeria | [16] |
|  | Ground water | | | 0.042 | 33.871 | 0.03387 | 1.1140 | 0.001114 |  |  |
|  | Sachet water | | | 0.001 | 0.806 | 0.00081 | 0.0265 | 2.65252E-05 |  |  |
|  | WWTPs Eff | | | 294.00941 | 237,104.363 | 237.10436 | 7,798.66 | 7.79866 | France | [12] |
|  | Guadiana basin | | | 4.806 | 3,875.806 | 3.87581 | 127.480 | 0.12748 | Portugal | [4] |
|  | Hospital wastewater | | | 0.008 | 6.452 | 0.00645 | 0.2122 | 0.000212 | Tunisia | [11] |
|  | WWTP | | Inf | 0.011 | 8.871 | 0.00887 | 0.2917 | 0.000292 |  | [11] |
|  |  |  | Eff | 0.07 | 56.452 | 0.05645 | 1.8567 | 0.0018567 |  |  |
|  | Coastal water | | | 0.023 | 18.548 | 0.01855 | 0.6101 | 0.0006101 |  | [11] |
| Ibuprofen | Surface water | | | 2.74 | 1,838.926 | 1.83893 | 65.8654 | 0.0658654 | Nigeria | [16] |
|  | Ground water | | | 2.25 | 1,510.067 | 1.51007 | 54.0865 | 0.0540865 |  |  |
|  | Sachet water | | | 0.05 | 33.557 | 0.03356 | 1.2019 | 0.0012019 |  |  |
|  | Guadiana basin | | | 3.161 | 2,121.476 | 2.12147 | 75.9856 | 0.0759856 | Portugal | [4] |
| Ketoprofen | WWTPs Eff | | | 255.1017 | 133,561.1 | 133.5611 | 966.2943 | 0.9662943 | France | [12] |
|  | Guadiana basin | | | 0.3214 | 168.272 | 0.16827 | 1.2174 | 0.0012174 | Portugal | [4] |
|  | Hospital wastewater | | | 18.1 | 9,476.439 | 9.47644 | 68.5606 | 0.0685606 | Tunisia | [11] |
|  | WWTP | Inf | | 3.3 | 1,727.748 | 904.5805 | 12.5 | 0.0125 |  | [11] |
|  |  | Eff | | 0.79 | 413.613 | 216.5511 | 2.9924 | 0.0029924 |  |  |
|  | Coastal water | | | 0.076 | 39.791 | 0.03979 | 0.2878 | 0.0002878 |  | [11] |
| Paracetamol (Acetaminophen) | Surface water | | | 12.43 | 11,098.214 | 11.09821 | 2.7869 | 2.79E-03 | Nigeria | [16] |
|  | Ground water | | | 0.188 | 167.857 | 0.16786 | 0.0422 | 4.22E-05 |  |  |
|  | Sachet water | | | 0.011 | 9.821 | 0.00982 | 0.0025 | 2.47E-06 |  |  |
|  | Nairobi river | | | 31.003 | 27,681.25 | 27.68125 | 6.9513 | 6.95E-03 | Kenya | [5] |
|  | WWTPs | | | 246.1237 | 219,753.304 | 219.7533036 | 55.1847 | 5.52E-02 | France | [12] |

**C**

| Types of waters | | The present study | | ECOSAR V2.2 | | Sampling location | References |
| --- | --- | --- | --- | --- | --- | --- | --- |
|  |  | **RQmix** | **TU** | **RQmix** | **TU** |  |  |
| Surface water | | 13,098.431 | 13.0984 | 73.957 | 0.07396 | Nigeria | [16] |
| Ground water | | 1,711.795 | 1.71179 | 55.243 | 0.05524 |  |  |
| Sachet water | | 44.1849 | 0.04418 | 1.231 | 0.00123 |  |  |
| Guadiana basin | | 6,165.555 | 6.16555 | 204.683 | 0.20468 | Portugal | [4] |
| WWTPs | | 590,418.766 | 590.4187 | 8,820.137 | 8.82014 | France | [12] |
| Hospital wastewater | | 9,482.891 | 9.48289 | 68.773 | 0.06877 | Tunisia | [11] |
| WWTP | Inf | 1,736.619 | 904.5893 | 12.792 | 0.01279 |  |  |
|  | Eff | 470.064 | 216.607 | 4.849 | 0.00485 |  |  |
| Coastal water | | 58.3389 | 0.05834 | 0.898 | 0.00089 |  |  |
| Nairobi river | | 27,681.25 | 27.6813 | 6.951 | 0.00695 | Kenya | [5] |
